# A Six Months Follow-Up on Children Less Than 6 Years Old in Contact With Smear Positive Tuberculosis Patients, Varamin City, Tehran, Iran

**Published:** 2011

**Authors:** Zohreh Aminzadeh, Rahim Taghizadeh Asl

**Affiliations:** 1 Associate Professor of Infectious Diseases, Shaheed Beheshti Medical University, Loghman Hakim Hospital, Infectious Diseases & Research Center, Iran; 2 MD

**Keywords:** Tuberculosis, Contacts, Follow-up, Chemoprophylaxis

## Abstract

**Objectives:**

Current international guidelines recommend 6-9 months of Isoniazid (INH) preventive chemotherapy to prevent the development of active tuberculosis in children exposed to smear positive tuberculosis (TB) patients. The aim of the present study was to evaluate the adherence to a six-month of supervised INH prophylaxis program and outcome in children with household exposure to an adult pulmonary tuberculosis index case in Varamin city, Tehran, Iran.

**Methods:**

This cross sectional study was conducted among household contacts in Varamin city, between 1997- 1998. All children <6 years old with a household contact with an adult pulmonary tuberculosis index case were screened for tuberculosis and given supervised INH preventive chemotherapy once active tuberculosis was excluded as planned. Adherence and outcome were monitored.

**Results:**

In total, 31 index cases and 128 household contact cases were identified; 23 (18%) children <6 years old experienced household exposure, who were fully evaluated. two children were treated for active tuberculosis and 15 (12%) children received preventive chemotherapy. All children completed 6 months of supervised INH prophylaxis with normal clinical examination in 3 and 6 months after beginning INH prophylaxis. No side effects (peripheral neuropathy or liver damage) were reported or observed within this study.

**Conclusions:**

Strategy of six months of supervised INH chemoprophylaxis is successful, particularly in children who are at a high risk to progress to disease following exposure.

## INTRODUCTION

It is estimated that 1.6 million People die due to tuberculosis every year.^1^ Transmission of disease continues until cases are detected and correctly treated.^2^ Children who are in close contact with drug-susceptible adult pulmonary TB have a high risk of being infected and developing the active disease.^3^ It is generally accepted that 30 to 50% of household contacts of adults with infectious forms of pulmonary TB will cause infection.^3^ The risk for young children with untreated infection to develop TB is up to 43% in children <1 year of age and about 24% for children 1 to 5 years old.^3^ Currently the World Health Organization (WHO) and the International Union against Tuberculosis and Lung Disease (IUATLD) recommend that all children <5 years of age who are in household contact with a sputum smear positive index case, should be actively traced and screened for tuberculosis.^4^,^5^ The purpose of this study was to evaluate the adherence to a six-month supervised INH prophylaxis program and outcome in children with household exposure to an adult pulmonary tuberculosis index case in Varamin city.

## METHODS

The study was a descriptive cross sectional one that was conducted among children with household contacts in Varamin city, between 1997 and1998. All children < 6 years old who were in close household contact with an adult pulmonary tuberculosis index case were screened to detect tuberculosis by researchers and given supervised INH prophylaxis once active tuberculosis was excluded. An index case was defined as an individual >15 years old with a positive smear of sputum for M tuberculosis. Childhood contacts were defined as children with 6 years of age or less living and sleeping in the same house or group of clustered houses on the same residential site as the index case for at least 1 month. Parents were asked to take these children to visit for initial and follow-up evaluations, even if the parents considered the child to be asymptomatic. All the children in these households were seen. Contacts were screened by clinical examination and tuberculin skin test. If there was a positive (>5mm) tuberculin skin test with normal clinical examination, supervised INH preventive chemotherapy was began for 6 months. If tuberculin skin test was negative (< 5 mm) with normal clinical examination, duration of supervised INH preventive chemotherapy was 3 months. After that, tuberculin skin test was repeated. If the repeated tuberculin skin test was positive (> 5mm), the supervised INH preventive chemotherapy was continued to complete a six-month coarse. But, if the repeated tuberculin skin test was negative, INH preventive chemotherapy was stopped. Contacts were screened by clinical examination in third and sixth month while they were taking INH chemotherapy. Obtained data were analyzed using SPSS (version 11.5) and descriptive statistical method.

## RESULTS

Totally, 31 patients with smear-positive pulmonary TB were considered as index cases. A total of 128 close contacts with index cases were identified, that 23 (18%) of them were children <6 years old, who were fully evaluated. Two children were treated for tuberculosis and 15 (12%) children were supposed to receive preventive chemotherapy. Positive smear results in index cases were as follow: 19 cases, 1+; 5 cases, 2+; 7 cases, 3+. Finally, 15 children needed INH prophylaxis, 3 children were removed due to their families immigration. Nine of 12 (60%) children were Afghan, 13 %^2^ were Iranians and 7 % (1) was Iraqi. Four children (33%) showed a positive (>5mm) tuberculin skin test before INH prophylaxis and 8 children (67%) showed a negative (<5mm) tuberculin skin test before INH prophylaxis. Children with negative test had a repeat test after 3 months of INH prophylaxis that there was a positive (>5mm) tuberculin skin test present in 5 children (63 %) but tuberculin skin test continued to be negative in three children (37%). Supervised INH preventive chemotherapy was continued in 5 children with positive skin test to complete six- month coarse. INH preventive chemotherapy was stopped in children with a negative (<5mm) repeated tuberculin skin test. All children who received INH preventive chemotherapy, completed 6 months of supervised INH prophylaxis with normal clinical examination in third and sixth month after beginning of INH prophylaxis. No side effects (peripheral neuropathy or liver damage) were reported or observed within this study.

## DISCUSSION

It is generally accepted that 30-50% of all household contacts with known cases of TB adults will have a positive tuberculin skin test.^3^ In this study, thirty three percent of household contacts <5 years of age with index cases had positive tuberculin skin test during follow-up.

In the present study, there was conversion of tuberculin skin test (negative to positive) in 63% of children after 3 months of receiving INH prophylaxis drugs; it was more than results of Johnsen et al. study.^6^ Johnsen study showed a conversion rate of 6.7% possible explanations are that all of the children in his study had a contact with smear-positive adult index cases.

Isoniazid was given to almost all children either as chemoprophylaxis for 6 months, and adherence to chemoprophylaxis was good. It does not agree with Van et al. study.^7^ In Van study, adherence to unsupervised chemoprophylaxis was poor. Adherence to a 3-month chemoprophylaxis regimen of isoniazid and rifampicin (3HR) was significantly better than adherence to a 6-month chemoprophylaxis regimen of isoniazid only.^7^

Simon Schaaf results^8^ suggested that following children who are in close household contact with adults with multi drug resistant pulmonary TB to detect the development of disease can be limited to 12 months, as only a minority of children would develop disease after this period. In our study, clinical examination of enrolled children in third and sixth month after beginning INH prophylaxis had been done and fortunately no new tuberculosis disease was found.

## CONCLUSION

Contact control and source-case investigations should be emphasized for TB control. However, considering the high incidence of infection and disease in our study, we believe that giving chemoprophylaxis should be the preferred management in contacted children with positive smear index cases. In areas with a high burden of disease and in poorly resourced countries where treating smear-positive TB cases is the priority, 6 months of directly observed chemoprophylaxis is appropriate.
